# The association of quality of life with potentially remediable disruptions of circadian sleep/activity rhythms in patients with advanced lung cancer

**DOI:** 10.1186/1471-2407-11-193

**Published:** 2011-05-23

**Authors:** James F Grutsch, Carol Ferrans, Patricia A Wood, Jovelyn Du-Quiton, Dinah Faith T Quiton, Justin L Reynolds, Christine M Ansell, Eun Young Oh, Mary Ann Daehler, Robert D Levin, Donald P Braun, Digant Gupta, Christopher G Lis, William JM Hrushesky

**Affiliations:** 1 Cancer Treatment Centers of America® at Midwestern Regional Medical Center, 2520 Elisha Ave. Zion, IL 60099, USA; 2 School of Medicine University of South Carolina, 6311 Garners Ferry Road, Columbia, SC 29209, USA; 3 School of Public Health, University of South Carolina, 800 Sumter Street, Columbia, SC 29208, USA; 4 University of Illinois, School of Nursing, 845 South Damen Avenue, Chicago, IL 60612 USA; 5 University of Illinois, School of Public Health, 1603 W. Taylor Street, Chicago, IL 60612, USA

## Abstract

**Background:**

Cancer patients routinely develop symptoms consistent with profound circadian disruption, which causes circadian disruption diminished quality of life. This study was initiated to determine the relationship between the severity of potentially remediable cancer-associated circadian disruption and quality of life among patients with advanced lung cancer.

**Methods:**

We concurrently investigated the relationship between the circadian rhythms of 84 advanced lung cancer patients and their quality of life outcomes as measured by the EORTC QLQ C30 and Ferrans and Powers QLI. The robustness and stability of activity/sleep circadian daily rhythms were measured by actigraphy. Fifty three of the patients in the study were starting their definitive therapy following diagnosis and thirty one patients were beginning second-line therapy. Among the patients who failed prior therapy, the median time between completing definitive therapy and baseline actigraphy was 4.3 months, (interquartile range 2.1 to 9.8 months).

**Results:**

We found that circadian disruption is universal and severe among these patients compared to non-cancer-bearing individuals. We found that each of these patient's EORTC QLQ C30 domain scores revealed a compromised capacity to perform the routine activities of daily life. The severity of several, but not all, EORTC QLQ C30 symptom items correlate strongly with the degree of individual circadian disruption. In addition, the scores of all four Ferrans/Powers QLI domains correlate strongly with the degree of circadian disruption. Although Ferrans/Powers QLI domain scores show that cancer and its treatment spared these patients' emotional and psychological health, the QLI Health/Function domain score revealed high levels of patients' dissatisfaction with their health which is much worse when circadian disruption is severe. Circadian disruption selectively affects specific Quality of Life domains, such as the Ferrans/Powers Health/Function domain, and not others, such as EORTC QLQ C30 Physical Domain.

**Conclusions:**

These data suggest the testable possibility that behavioral, hormonal and/or light-based strategies to improve circadian organization may help patients suffering from advanced lung cancer to feel and function better.

## Background

Oscillators with a circadian (about 24-hr) period synchronize and co-ordinate the behavior of biological systems at all levels of biological complexity, to specific phases of the geophysical day [1-7]. Desynchronizing an individual's circadian system with the phase of the geophysical day adversely affects health, mental and physical, as well as psychological function and health-related quality of life [8-10]. Cancer patients experience a deterioration in the robustness (amplitude) and day-to-day phase stability of their rest/activity rhythms that faithfully and quantitatively reflect cancer stage, patient performance status, and prior treatment status [10-15]. Consequently, cancer patients develop specific symptom clusters pathognomonic of a disrupted circadian organization, including poor nighttime sleep quality, depressed mood, and increased anxiety; daytime fatigue and lethargy; and anorexia, early satiety, and diminished taste sensation [15-21]. The occurrence of these sickness behaviors among individuals suffering circadian disruption strongly suggests that a disrupted circadian organization adversely affects a patient's quality of life. Lung cancer is the most prevalent lethal cancer in the developed and developing world. However, there have been few investigations on the relationship between lung cancer patients' circadian organization and their quality of life [10,15].

Linking changes in a patients' circadian organization to their quality of life is a complicated process. Quality of life is measured using a diverse assortment of outcomes that range across physical, psychological, social, and spiritual domains. Furthermore, for each assessed quality of life category there are a variety of different instruments. Physical domain assessment may measure symptoms, or the patient's ability to meet the challenges of daily life, or adjustment to illness [22]. Quality of life is, thereby, a multidimensional concept, within which each specific category has a range of unique attributes. Wilson and Cleary [23] have developed a quality of life model that expresses the relevant logical connections among several quality of life measurements along a sequential pathway. This pathway establishes that physiological disturbances produce symptoms in patients that change their functional health, which in turn affects the patients' perceptions of their general health, which in turn contributes directly to their perceptions of their overall quality of life.

We measured circadian organization non-invasively using wrist actigraphs [24-28]. We also implemented the EORTC QLQ-C30 to measure the existence and intensity of the patient's symptoms (symptom status) as well as the patient's perception of his or her functional health [29,30]. We also utilized the Ferrans/Powers Quality of Life Index (QLI) to measure the patient's level of satisfaction with how well their current life achieves an acceptable level of well being [22,31].

## Methods

### Patients

Patients participating in this investigation were recruited for a clinical trial that was conducted concurrently at Cancer Treatment Centers of America^® ^(CTCA) at Midwestern Regional Medical Center (MRMC) in Zion, Illinois and the WJB Dorn Veterans Medical Center (VAMC) in Columbia, South Carolina from June 2002 to April 2006. Patients between the ages of 18 and 80 who had a pathologically confirmed diagnosis of stage III or IV non-small cell lung cancer (NSCLC) and an Eastern Cooperative Oncology Group (ECOG) performance status of 0, 1, or 2 were eligible. Untreated patients and patients who had failed one prior chemotherapy treatment regimen were eligible. All patients signed an Informed Consent indicating that they were aware of the investigational nature of the study. The Institutional Review Boards at MRMC and VAMC approved the study. This current report is based only upon baseline data obtained at initial enrollment.

### Control Subjects

Our control database for actigraphic parameters is comprised of 3- to 7-day actigraphy measurements from 35 adults, aged 20 to 50 years, each having no known disease (Action-W2 database, AMI, NY, USA). EORTC QLQ-C30 Reference Population data is from a survey of 1,313 newly diagnosed lung cancer patients with stage III and IV disease,^32 ^while the EORTC QLQ-C30 General Population data is from a survey of 1,965 randomly selected subjects in Norway aged eighteen to ninety-three years [33]. Ferrans/Powers QLI Health/Functioning General Population data is taken from 339 subjects, drawn randomly from a telephone directory representing urban, suburban, and rural areas in the Midwestern United States, who completed the QLI questionnaire (C. Ferrans, Feb 2009).

### Protocol Summary

All patients agreed to complete their baseline quality of life questionnaires before undergoing their initial chemotherapy treatment cycle for this trial. At MRMC, actigraphy was performed in the inpatient setting immediately before (24-48 hours) and during their first chemotherapy cycle. VAMC actigraphy was obtained in the patients' domestic setting, prior to their initial cycle of chemotherapy.

## EORTC QLQ-C30 and LC-13

The thirty question European Organization for Research and Treatment of Cancer Quality of Life Questionnaire (EORTC QLQ-C30) and the thirteen question Lung Cancer Questionnaire (LC-13), were developed by clinical trialists to measure lung cancer patients' ability to fulfill the routine activities of daily life.^29 ^On the EORTC QLQ-C30, five function domains are used to measure physical, role, emotional, social, and cognitive functions. In addition, the EORTC QLQ-C30 measures the following symptom items; fatigue, nausea/vomiting, loss of appetite, pain, dyspnoea, insomnia, and financial.

On the LC-13, has both multi-item and single-item measures of lung cancer associated symptoms. Six of the questions measure the four lung cancer associated symptoms items: coughing, haemoptysis, dyspnoea and pain. The other questions measure the side-effects from conventional chemo- and radiotherapy: hair loss, neuropathy, sore mouth and dysphagia. The possible scores range from 0 to 100. Higher scores in the global and functional domains indicate better Quality of Life and lower scores in the symptom items indicate better Quality of Life.

### Ferrans and Powers Quality of Life Index (QLI) Cancer Version III

The Ferrans and Powers Quality of Life Index (QLI) differs from the EORTC QLQ-C30 in that it elicits information quantifying each patient's satisfaction or dissatisfaction with various aspects of life and thus assesses the individual's feelings of emotional well-being and capacity to enjoy life. Consequently, this instrument evaluates how cancer has affected, both positively and negatively, the patient's well being in terms of their own personal values [22]. The QLI Cancer Version III consists of two parts. Part one requires the patient to describe satisfaction levels with 33 aspects of life. Part two requires the patient to rate the importance of the same 33 aspects of his or her life. The QLI produces an overall quality of life score and subscale scores on four specific domains: health and functioning, social and economic, psychological and spiritual, and family. The scores range from 0 to 30, with higher scores indicating greater satisfaction with life. Thus, the EORTC-QLQ C30 and the QLI provide distinct, unique, and complementary insights into different aspects of each patient's quality of life [34].

### Actigraphy Measurements of Rest-Activity Cycles

A watch-like wrist actigraph, worn on the non-dominant wrist, was used to record a patient's movement patterns continuously over a four to seven day span (Ambulatory Monitoring, Inc, AMI, Ardsley, NY USA). Internal motion sensors (accelerometers) capture patient movement data, measured as the number of accelerations per minute. These data are transferred to a computer for analysis to produce a report containing parameters of activity during each sleep and wake period, detailing their extent, timing, duration and other characteristics [35]. Actigraphy data were recorded in the hospital setting at MRMC in the four to seven days immediately prior to and during the first treatment, while these data were obtained in the patient's home for VAMC patients during the week prior to the first treatment.

### Determination of Presence and severity of Chronic Obstructive Pulmonary Disease (COPD)

Chronic Obstructive Pulmonary Disease (COPD) is common among lung cancer patients and, thereby, a potential confounding variable for this investigation of quality of life outcomes and circadian time structure. All VA patients were assessed clinically and with pulmonary functions for the presence of COPD. Its severity was graded according to the Spirometric Classification of COPD severity, by reference to percent of predicted forced expiratory volume in one second (FEV_1_). Thirty to fifty percent of predicted FEV_1 _is considered severe; moderate is 50 to 80%; and mild COPD is greater than 80% of predicted FEV_1. _No such data were available for inpatients.

### Statistical Analysis

Descriptive statistics such as mean and standard error were computed for continuous variables and actigraphy endpoints to describe the average and variability of values across the population. Frequency and percentages were computed for qualitative factors such as sex and quality of life outcomes. Both parametric (Analysis of Variance) and non-parametric analyses were used to determine time of day differences among factor levels (SAS v 9.1, Cary, NC) and their shapes across (circadian) time of day. For four to seven days, an actigraphy watch recorded the number of accelerations per minute. These data were translated into polysomnographically-validated sleep/activity parameters through the Act Millenium and Action W2 software (Ambulatory Monitoring, Inc). Cosinor analysis was done on these sleep/activity patterns in order to assess the circadian characteristics of sleep and activity among these lung cancer patients. Cosinor analysis provides three standard parameters: mesor - the average activity over the 24-hr period, amplitude - peak to nadir difference and acrophase - the time of daily peak activity. We also computed measures of circadian rhythm robustness and day-to-day stability (circadian quotient = amplitude/mesor), peak activity (mesor + amplitude) and 24 hour autocorrelation for each patient. We further measured circadian indices of the daily timing of sleep and activity, such as the ratio of amount of activity during the day to nighttime activity, night/day sleep balance that is the amount of nighttime sleep relative to daytime sleep. Inpatient and outpatient differences were tested using t-test for quantitative characteristics while chi-square was used for categorical factors. The relationships between quality of life domain values and actigraphy measurements were measured using the Spearman rank correlation test.

## Results

### Patient Characteristics

84 patients were enrolled and consented at two sites. One site studied patients only in the outpatient home environment while the second site studied them only as inpatients. There were differences in demographic and clinical status in participants by site. All 42 outpatients were males while 19 of 42 inpatients were female. Outpatients were older on average with a mean age of 66 compared to the inpatients' mean age of 57 years (Table 1). More inpatients had Stage IV disease (74.4%) than outpatients (47.6%).

**Table 1:** Distribution of patient demographic and clinical variables by site

Demographic/Clinical^a^	Inpatients (n = 42)	Outpatients (n = 42)	Site Difference (χ^2^, p)^b^
Age in years (Mean; Range)	57 (40-78)*	**66 (47-94)**	4.0, < 0.01^**c**^
Sex (M:F)	23:19*	**42:0**	24.6, < 0.01
Cancer Stage (IIIA&B: IV)	10:32	9:33	2.1, 0.36
Prior Therapy (Yes:No)	**21:20**^d*^	**10:32**	6.7, 0.01
WHO ECOG (0:1:2)	17:18:07	13:24:04^d^	2.2, 0.33
COPD (No:Mild:Mod:Severe)^c^		14:7:13:8	

^a^Values are numbers of patients except for Age. ^b^Based on chi-square test. ^c^Based on t-test. ^d^One patient at MRMC lacked documentation of prior therapy, while another patient at VAMC lacked documentation on ECOG scale. *Significant difference at p < 0.05 compared to outpatients.

50% and 26% of inpatients and outpatients, respectively, were experiencing recurrence of disease after first line systemic treatment failure. The median time between terminating first line therapy and the administration of actigraphy in the ten outpatients was 51.6 months (range 7.9 to 78 months), but for inpatients it was 3.5 months (0.8 to 33.5 months).

### Assessment of Patient Evaluability

Although all patients agreed to wear the actigraphs, twelve actigraphs were worn for less than 48 hours and/or had frequent missing observations due to instrument malfunction. Four patients failed to respond fully to the questionnaires, so we have full actigraphy and full questionnaire data for 68 of these 84 patients.

### Quality of Life

Both inpatients and outpatients reported lower scores for all EORTC-QLQ-C30 functional domains when compared to population-based controls (Table 2). The differences in domain scores between these lung cancer patients and population-based controls were large, ranging from 16 to nearly 30 points. Both inpatients and outpatients reported scores that were 20 to 30 points higher (worse) than the population based controls in the symptom items of fatigue, loss of appetite, insomnia, and pain. Our advanced lung cancer patients, as expected, also scored 10 to 20 points higher (worse) on these symptom items than the EORTC reference population of newly diagnosed lung cancer patients with stage III and IV disease. Despite various demographic and clinical differences between inpatients and outpatients, only two of 20 symptom categories, fatigue (p = 0.0187) and shortness of breath (p < 0.001), achieved statistical significance between our two patient groups, indicating the profound leveling effect of advanced non-small cell lung cancer *vis a vis *their most common and devastating symptoms.

**Table 2:** Distribution of EORTC QLQ-C30 domain and symptom item scales by site

Domain	Study Population (mean ± se)			General Population^c ^(mean)	Reference Population^d ^[mean (SD)]
	All Patients	Inpatients	Outpatients		
**Global Score**^**a**^	50.78 ± 3.15	49.80 ± 4.84	51.96 ± 3.86	-	54.7 (23.8)
**Functional Scales**^**a**^					
Physical	64.53 ± 3.15	73.33 ± 4.22*	53.92 ± 4.12*	89.9	65.9 (25.6)
Role	54.44 ± 4.28	60.57 ± 6.01	47.06 ± 5.91	83.3	55.5 (34.5)
Emotional	59.52 ± 3.31	59.96 ± 4.24	58.99 ± 5.29	82.8	67.3 (24.1)
Cognitive	70.22 ± 3.18	76.02 ± 4.15	63.24 ± 4.71	86.5	81.6 (22.7)
Social	55.33 ± 3.77	51.22 ± 5.35	60.29 ± 5.22	85.8	69.8 (30.3)
**Symptom Scales**^**b**^					
Fatigue	52.00 ± 3.55	45.26 ± 4.63*	60.29 ± 5.22*	28.8	44.2 (27.5)
Nausea	15.78 ± 2.53	14.63 ± 3.30	17.16 ± 3.95	4	10.8 (19.1)
Pain	53.33 ± 4.38	48.37 ± 6.10	59.31 ± 6.22	20.5	34.7 (32.3)
**Single Items**^**b**^					
Dyspnoea	46.22 ± 3.79	36.58 ± 4.33*	57.84 ± 6.02*	14.3	40.7 (32.2)
Insomnia	44.00 ± 3.91	39.84 ± 4.97	49.02 ± 6.17	20.4	34.8 (33.4)
Appetite Loss	39.11 ± 3.97	33.33 ± 4.94	46.08 ± 6.30	7.5	31.1 (34.6)
Constipation	29.33 ± 3.90	26.83 ± 4.83	32.35 ± 6.37	10.4	22.2 (31.7)
Diarrhea	7.11 ± 2.03	5.69 ± 2.83	8.82 ± 2.92	9.4	7.3 (18.1)

^a^Higher scores indicate better daily function, thus better quality of life. ^b^Lower scores indicate fewer symptoms, thus better quality of life. ^c^EORTC general population of 1,965 randomly selected subjects ages 18 to 93 years (Hjermstad, et. al. *J Clin Oncol *1998)., ^d^EORTC reference population of 1,313 lung cancer patients with stage III and IV disease (Scott, et. al. *EORTC Reference Manual *2008). *Inpatients versus Outpatients are significantly different, p < 0.05.

Our patients' mean score of 16.23 in the Ferrans/Powers QLI health/functioning domain is nearly eight points lower (worse) than the population-based mean score. This is some two standard deviations below mean scores from a general population survey. Patients were very dissatisfied with their health. Interestingly, however, the other three QLI domains (social/economic, psychological/spiritual well being, and family) did not differ at all from the population-based scores (Table 3).

**Table 3:** Distribution of Powers and Ferrans QLI domain scores by site

Domain	Study Population (mean ± se)			General Population^b ^[mean (SD)]
	All Patients	Inpatients	Outpatients	
Health & Functioning^a^	16.23 ± 0.72*	15.13 ± 1.14	17.55 ± 0.0.73	23.19 (4.47)*
Social/Economic^a^	21.15 ± 0.46	21.21 ± 0.58	21.08 ± 0.74	21.83 (4.11)
Psychological/Spiritual^a^	21.57 ± 0.71	21.04 ± 0.88	22.21 ± 1.16	22.95 (5.21)
Family^a^	23.22 ± 0.60	24.50 ± 0.75	21.68 ± 0.89	25.60 (4.49)
Overall Quality of Life^a^	19.61 ± 0.48	19.28 ± 0.69	20.02 ± 0.66	23.00 (4.04)

^a^Higher scores indicate greater satisfaction with life. ^b^Powers and Ferrans QLI General Population is taken from a database of 339 randomly selected subjects ages 18 and above (C. Ferrans, Feb 2009). *Study Population scores are markedly lower than General Population scores.

### Actigraphy

**Figure 1:**
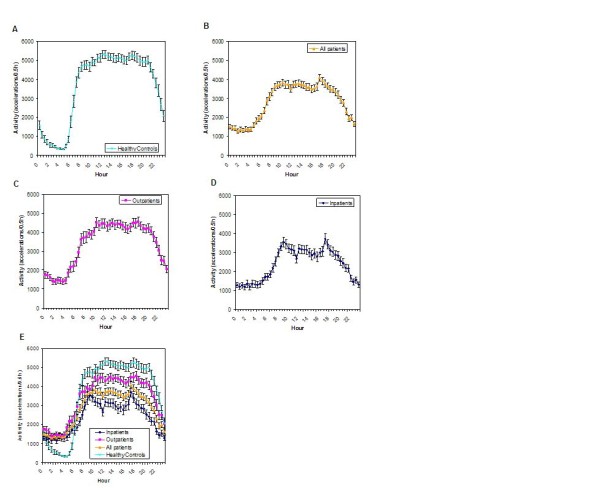
**Comparison of the circadian activity rhythm of healthy controls and lung cancer patients**. Healthy controls (**A**) show a more robust circadian activity rhythm compared to lung cancer inpatients and outpatients combined (**B**). Outpatients (**C**) have better circadian organization of sleep/activity compared to the hospitalized group (**D**). Concurrent plot of all groups (**E**) shows that peak daytime activity is compromised in all lung cancer patients and especially among hospitalized ones. Nightly sleep is, however, markedly and identically disturbed among lung cancer patients regardless of where it is measured.

Actigraphic parameters of lung cancer patients were found to be grossly abnormal when compared to the healthy control population. Healthy individuals show a more robust 24-hour activity rhythm while lung cancer patients show a flatter curve (Figure 1). Cancer patients were 30% less active and napped at least three times longer than the controls during the putative wake/activity period (Table 4). During the nightly sleep span, lung cancer patients had significantly more and longer waking episodes than controls (Table 4). No gender differences were found.

**Table 4:** Actigraphic sleep-activity characteristics of non-small cell lung cancer patients compared to normal individuals during the putative wakefulness and during the putative sleepfulness

Parameters	All patients	Inpatients	Outpatients	Healthy Controls
N	68	35	33	35
**Daytime Activity**				
				
Mean Activity (accel/min)	126.9 ± 4.9	**111.7 ± 7.1**	**143.0 ± 5.6**	182.6 ± 25
**Wake Minutes**	797.5 ± 26	**714.2 ± 36**	**885.8 ± 31**	947.1 ± 11
**Sleep Minutes**	208.8 ± 18	**241.3 ± 25**	**174.4 ± 24**	46.5 ± 6.9
**% Sleep**	20.9 ± 1.8	**25.8 ± 2.8**	**15.6 ± 1.9**	4.7 ± 0.7
**Duration of Longest Sleep (min)**	43.0 ± 2.8	**45.4 ± 4.0**	**40.5 ± 3.9**	23.6 ± 0.6
				
**Nighttime**				
				
**Wake Minutes**	95.0 ± 8.8	**97.5 ± 12.2**	**92.3 ± 12.9**	31.1 ± 3.6
**Sleep Minutes**	284.0 ± 18.3	**300.5 ± 24.8**	**266.4 ± 27.2**	417.89.4
**% Sleep**	72.5 ± 2.0	**73.3 ± 2.6**	**71.6 ± 3.08**	93.0 ± 0.8
**Sleep Efficiency (%)**	79.8 ± 1.7	**80.8 ± 2.4**	**78.7 ± 2.5**	95.9 ± 0.7
**Duration of Longest Sleep**	91.7 ± 7.4	**100.3 ± 11.8**	**82.5 ± 8.6**	225.6 ± 17
**Sleep Latency (min)**	20.8 ± 2.5	**18.1 ± 2.9**	**23.7 ± 4.0**	12.1 ± 6.9

*Inpatient vs. Outpatient difference is significant for all parameters (t-test, p < 0.05).

Actigraphy characterizing the putative daily wake span and the overall circadian organization differed by site (Table 4, Figure 1). Outpatients were more active during the day and consolidated activity and sleep better compared to hospitalized inpatients. The sleep phase actigraphy parameters at both sites, however, were indistinguishable. These prominent site specific differences in actigraphy collection protocol required that all such data be analyzed by site. The most obvious ubiquitous and severe differences across the groups of normal controls and lung cancer patients include uniformly poor sleep among all lung cancer patients and decreasing daytime activity which is most severe, as might be expected, among hospitalized patients.

### Correlation of Quality of Life and Actigraphy among Inpatients

Among inpatients we only found several statistically significant associations with actigraphy parameters. We found that the QLI Health/Functioning domain scores correlate with the 24-hour activity autocorrelation, which measures day to day stability of the peak and trough timings of the daily activity/sleep pattern (r = 0.34, p = 0.05). Inpatients with the most stable circadian time structures of the daily activity pattern express the highest level of satisfaction with their health. Those who are most disrupted have much worse satisfaction with their health. Two EORTC symptom items correlated strongly with activity circadian rhythm parameters; insomnia severity correlates negatively with 24-hour autocorrelation, the day-to-day reproducibility of peak and trough activity (r = -0.48, p = 0.003), and loss of appetite correlates with decreased peak daily activity (r = -0.41, p = 0.005) and Circadian Quotient, a measure of the strength of the circadian rhythm compared to noise or other high frequency rhythms (r = 0.4, p = 0.015). Circadian activity/sleep rhythm disruption is reflected in or caused by nocturnal insomnia and those patients with most decreased daily activity (ie. greatest daytime fatigue) have the poorest appetite, even in the hospital setting.

### Relationships between QLI and Actigraphy among Outpatients

**Figure 2:**
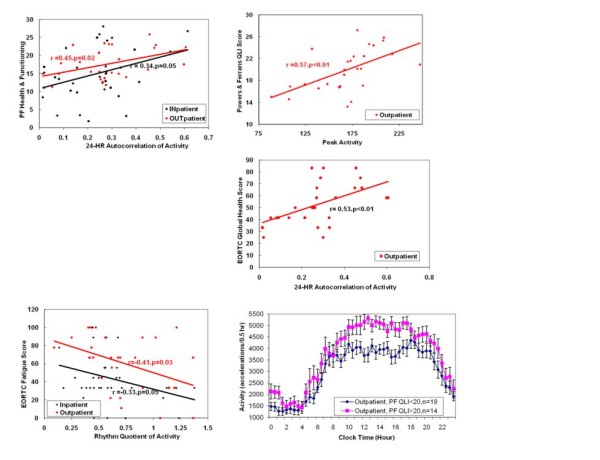
**Relationship between quality of life and circadian organization among inpatients and outpatients**. Lung cancer patients who are most quiet in the nighttime and active during the day (highest circadian Rhythm Quotient) are the least fatigued, both in hospitalized and at home settings (**A**). The more stable the day-to-day pattern of daily activity and nighttime sleep (24-hour Autocorrelation), the better the overall ability to fulfill daily functions among outpatients (**B**), and the greater the patient's satisfaction with his/her health among inpatients and outpatients (**C**). The greater the peak activity, the greater the patient's overall satisfaction with life among outpatients (**D, E**).

Among ambulatory outpatients with advanced lung cancer in their own homes, higher levels of daytime activity, as measured by the parameter "peak activity," is significantly associated with each of the five Power and Ferrans QLI domains: health/functioning(r = 0.51, p < 0.01), social/economic (r = 0.38, p = 0.048), psychological/spiritual (r = 0.45, p = 0.02), family (r = 0.45, p = 0.02), and overall QLI (r = 0.57, p < 0.01), see Figure 2 and Table 5. Robustness (largest peak to trough difference in daily activity or amplitude) of objectively measured daytime activity is reflective of all measured aspects of quality of life in the face of advanced lung cancer. The health/functioning domain also demonstrates a statistically significant positive relationship with 24-hour autocorrelation, or day-to-day circadian stability of peak and trough activity timings (r = 0.45, p = 0.02). Each Ferrans-Powers QLI domain exhibits a statistically significant relationship with the actigraphy parameter night-day sleep balance and quality of life scores in health/functioning (r = 0.39, p = 0.04), social/economic (r = 0.40, p < 0.04), psychological/spiritual (r = 0.45, p = 0.02), and family (r = 0.33, p = 0.10). The more robust the day/night difference between nocturnal and daytime activity levels, the better the patients score by each and every QLI measure.

**Table 5:** Statistically Significant (P < 0.05) Correlations between Quality of Life (QoL) Domains and Cosinor Parameters in Outpatients

QoL Domains	Mesor	Amplitude	Circadian Quotient	Rhythm Quotient	24-hr Correlation	Night-day Sleep Balance
**EORTC Symptom Items**						
						
Fatigue			-0.4	-0.41		-0.52
Pain				-0.39		
Loss of Appetite						-0.47
						
**EORTC Functional Domains**						
						
Social				.34		
Role						0.56
Cognitive						0.45
						
**EORTC Global Health item**					0.53	
						
**Ferrans-Powers QLI Domains**						
						
Health/Function	0.44	0.51			0.45	0.39
Social/Economic		0.38	0.39			0.40
Psychological Spiritual		.45		0.4		0.45
Family		0.45				

### Relationships between EORTC QLC 30 and Actigraphy-measured Circadian Function

Statistically significant correlations between EORTC QLQ C30 domains among outpatients were found. Global health exhibits a statistically significant positive association with circadian phase stability as reflected by the 24-hour autocorrelation (r = 0.53, p < 0.01), (see figure 2). The actigraphy parameter, night-day balance of time spent asleep and awake, shows a statistically significant association with the EORTC QLQ C30 domains role (r = 0.56, p < 0.01) and cognitive function (r = 0.45, p = 0.02).

### Circadian Organization and EORTC Symptom Items

Outpatient fatigue levels are associated with diminished robustness of the circadian quotient (r = -0.40, p = 0.04), rhythm quotient (r = -0.41, p = 0.03) and night-day balance of time spent asleep (r = -0.52, p < -.01). The more quantitatively robust the day-night activity/sleep measurement differences, the less fatigue these patients experience during each day. Rhythm quotient is also inversely associated with pain (r = -0.39, p = 0.04). The greater the pain, the less robust were day-night activity differences, objectively verifying that pain interferes with both sleep at night and activity during each day. Loss of appetite is likewise associated negatively with the actigraphy parameters night-day sleep balance (r = -0.47, p < 0.01). The more disordered the circadian organization, the more profound the cancer-associated loss of appetite.

Unlike the analysis for quality of life outcomes, there were no statistically significant associations found between actigraphy data and chronic COPD severity in this patient population with symptomatic advanced lung cancer.

### Relationships of Quality of Life Scores to one another and to Circadian Organization

**Figure 3:**
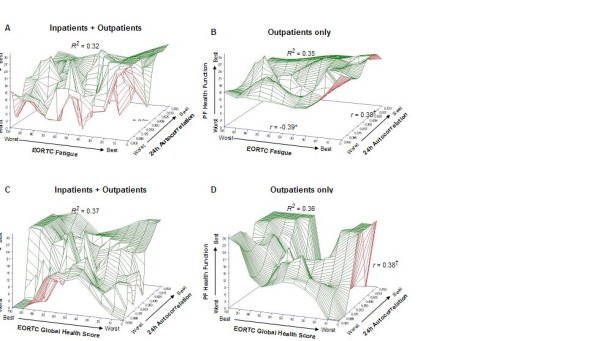
**The relationships among two measures of quality of life and circadian rhythm stability**. The greater the circadian rhythm in activity/rest, the greater the PF Health and Functioning; the lower the EORTC Fatigue Score (**A**) and the greater the EORTC Global Health Score (**C**) in the group as a whole. These relationships are especially clear among outpatients (**B, D**). *Significant *r*, *p *< 0.05. ^†^Marginal *r*, *p*=0.08.

Figure 3 shows the relationships among two measures of quality of life and circadian organization parameters. A robust circadian rhythm in activity/rest is associated with greater patient satisfaction with health/functioning, less fatigue, and better overall quality of life. These relationships, though present among all patients, are especially clear among outpatients where hospitalization does not mask usual circadian time structure.

## Discussion

Circadian organization optimizes organismal function at all levels of biological organization [4,39,40]. A faltering circadian organization generates symptoms that range from the fairly minor, such as jet lag, to life limiting disorders like depression, metabolic syndrome, cardiovascular or gastrointestinal disease, or cancer [41]. A breakdown of circadian organization can result in death in lab animals and may contribute to death in humans [39,42-45].

Cancer bearing degrades circadian organization, which diminishes an individual's health and capacity to meet the demands of daily life [10,46,47]. Apparently, a patient's health-related quality of life is adversely affected when the level of disruption of an individual's circadian organization exceeds a threshold [48].

Our actigraphy data show that non small cell lung cancer patients' circadian activity/rest rhythms are markedly and characteristically distorted when compared to apparently healthy individuals. Our advanced lung cancer patients' daily activity levels are much lower than population-based control data. Our patients took many prolonged daytime naps, while suffering many episodes of prolonged awakening for prolonged spans during each night. Similar findings have been reported for patients early stage breast cancer and metastatic colorectal cancer [52,53]. These patients also reported quality of life outcomes that were significantly lower than population based controls. Our patients' responses to the EORTC instrument reveal they were experiencing multiple symptoms and their capacity to meet the challenges of routine life was compromised. Our patients, like others with advanced cancer, were very dissatisfied with their health. Every patient self report for the QLI health/functioning domain fell within the bottom quartile as compared to the general population scores, indicating a uniformly high level of dissatisfaction with their physical health. On the other hand, their scores for the other three QLI domains, social/economic, family, and psychological/spiritual were indistinguishable from those of the general population. Our patients were emotionally and psychologically healthy, despite lethal cancer associated ill health. This finding illustrates the resilience of the human spirit, even in the face of lethal symptomatic disease and the value of asking questions relevant to the whole life of the cancer patient.

An interesting finding was the lack of a significant relationship between a self-reported insomnia among outpatients and any objectively measured actigraphy parameter. This finding is surprising considering that actigraphy data showed that virtually every patient's sleep was fragmented and unconsolidated and these patients' self reports of their sleep quality, as measured by a validated sleep questionnaire, was indistinguishable from insomniacs [49,50]. This *non sequitor *between universal objective signs of very poor sleep and the perception of insomnia among advanced lung cancer is interesting. It seems as though, in cancer patients as in the healthy elderly, the perception of nocturnal sleeplessness and the reality of it are at odds. Inpatients did report an association between an actigraphy parameter and insomnia. We can speculate that hospital routines involve a sudden increase in number of untoward nighttime awakenings that results in the patients feeling much sleepier than they did in the prior week. Similar findings have been reported for patients with metastatic colorectal cancer [52]. Two-thirds of the patients for whom it was measured suffered from mild, moderate or severe chronic COPD. There were no statistically significant relationships between the degree of dyspnea and any parameter of circadian function. These findings indicate that both lung cancer and COPD are associated with shortness of breath but that quality of life decrement is only associated with cancer symptoms and circadian disruption and not COPD severity, which is usually relatively stable and chronic.

Among outpatients, whose daytime activity levels were expectedly higher than inpatients, there were many clinically relevant and statistically significant associations between most parameters of circadian organization and cancer associated daytime fatigue that were masked, weakened or lost among inpatients. We conclude that all future studies of cancer-related quality of life and circadian organization should best be done in the patient's home, not in the hospital.

All Ferrans-Powers QLI domains, except Family, which had one statistically significant relationship, had multiple strong statistically significant relationships with cosinor and actigraphy parameters of circadian organization. Because of this sensitivity and relevance, we believe that future work should employ the Powers and Ferrans scales in addition to any other quality of life scales.

Superficially, advanced lung cancer patients appear to be suffering from non-resolving jet lag; disturbed as well as diminished non-restorative sleep; and excessive daytime sleepiness and nighttime wakefulness, daytime fatigue, problems with concentration and memory during the day, and loss of appetite, especially during the second half of the waking hours. We found many strong correlations between all Ferrans-Powers QLI domains of each individual and most parameters of that individual's circadian organization. Apparently a decaying circadian organization affects a patient's perception of their health and emotional/psychological well being before it actually reduces his/her capacity to fulfill these activities of normal life. This finding of the possible premonitory value of circadian status deserves careful additional quality of life study.

There are several limitations in this study. Our sample size may not be large enough to accommodate the number of comparisons made within this study. We unavoidably lacked, by virtue of the nature of our oncology practices and the VA setting of one of our groups, perfect age- and gender-matched comparisons to clearly evaluate the extent of the affects of sex and age upon circadian function of our cancer inpatients vs. outpatients. We did, however, obtain relevant actigraphy data upon 35 healthy controls aged 20 to 30 for comparison. While not a perfect control, this population provides reasonable comparison data. The most significant limitation may be that our statistical models are less than optimally sensitive to linking the degree of disorder in the patient's circadian system and advanced lung cancer symptoms [51].

Despite these very real limitations, our data and those of others indicate that among cancer patients, circadian organization is an integrative pathway involved in a variety of important pathophysiologic processes that prominently and quantitatively affect a variety of quality of life outcomes. The next step in this program is to determine whether restoring the robustness of circadian organization among advanced cancer patients is possible and whether doing so will reduce symptom intensity, improve function and overall satisfaction with their physical/emotional/psychological health, improve performance status and well being, and ultimately determine whether survival duration can be prolonged by improving the circadian organization of patients with advanced cancer.

## Conclusions

These data suggest the testable possibility that behavioral, hormonal and/or light-based strategies to improve circadian organization may help patients suffering from advanced lung cancer to feel and function better.

## Competing interests

The authors declare that they have no competing interests.

## Authors' contributions

JFG, CF, PAW, MAD, RDL, CGL and WJMH participated in the concept, design and data interpretation. JFG, JDQ, JLR, DPB, and WJMH were involved in the statistical analysis. JFG, JDQ, DG and WJMH were involved in the writing. DFTQ, JLR, CMA, EYO, MAD and RDL were involved in patient recruitment and data management. All authors read and approved the final manuscript.

## Appendix 1. Circadian Parameters Used

The following parameters described the activity phase of the daily circadian cycle: mean daily activity, mean duration of activity during wakefulness, mean duration of sleep during wakefulness, proportion of wakefulness spent sleeping, number of sleep episodes during wakefulness, and frequency of long naps. During the presumed sleep phase of the circadian cycle, the following parameters were evaluated: mean duration of wakefulness, number of sleep interruptions, proportion of sleep episode spent actually sleeping, and frequency of long duration of sleep. The number of accelerations per minute for a continuous 4-7 days was recorded through the actigraphy watch and was translated into sleep/activity parameters through the Act Millennium and Action W2 software (Ambulatory Monitoring, Inc.).

Rhythmometric analysis (using Chronolab v2) was carried out on these sleep/activity patterns in order to assess disruption and consolidation of sleep in lung cancer patients. Rhythmometric analysis fits a cosine curve to the circadian activity providing three standard parameters: mesor - the average activity over the 24-h period, amplitude-peak to nadir difference and acrophase - the time of peak activity. In addition to these parameters, peak activity (mesor+amplitude) to measure activity levels, the circadian quotient (amplitude/mesor) to characterize the strength of the circadian rhythm, and the rhythm quotient (A_24 h_/(A_4 h_+A_8 h_+A_12 h_)) were computed. A_4 h_, A_8 h_, and A_12 h _are the amplitudes of 4-hr, 8-hr and 12-hr cosine fits, respectively. Higher amplitudes indicate more robust rhythms but people who move vigorously would have higher amplitude, thus, the circadian quotient provides normalized values that would allow comparison between individuals [54,55].

We also looked at the dichotomy index (I < O), comparing amounts of activity when in bed and out of bed, which is significantly associated with colorectal cancer patients' quality of life and survival ^14^. I < O is the percentage of minutes or epochs during the putative sleep span with activity score that are less than the median activity during the putative wakefulness. Thus, high I < O reflects a marked rest/activity rhythm ^15 ^Further, circadian rhythms were assessed through spectral density analysis where 24-hour autocorrelations (r24) were computed. Autocorrelations theoretically can range from 0 to1. If a circadian variation is present, autocorrelations will increase around 24-hour and a more pronounced and stable day-to-day circadian rhythm will result in a higher autocorrelation at 24-hour.

## Pre-publication history

The pre-publication history for this paper can be accessed here:

http://www.biomedcentral.com/1471-2407/11/193/prepub
